# The Kantian account of mechanical explanation of natural ends in eighteenth and nineteenth century biology

**DOI:** 10.1007/s40656-022-00484-0

**Published:** 2022-03-08

**Authors:** Wim Beekman, Henk Jochemsen

**Affiliations:** Philosophy Group, Wageningen University & Research, Hollandseweg 1, 6706 KN Wageningen, The Netherlands

**Keywords:** Natural end, Kant, Organism, Explanation, Generation, Eighteenth and nineteenth century

## Abstract

The rise of the mechanistic worldview in the seventeenth century had a major impact on views of biological generation. Many seventeenth century naturalists rejected the old animist thesis. However, the alternative view of gradual mechanistic formation in embryology didn’t convince either. How to articulate the peculiarity of life? Researchers in the seventeenth century proposed both “animist” and mechanistic theories of life. In the eighteenth century again a controversy in biology arose regarding the explanation of generation. Some adhered to the view that life is a physical property of matter (e.g. Buffon), others saw living entities as the result of the development of pre-existing germs (e.g. Bonnet). Naturalists, lacked a convincing account that could guide their research. In interaction with leading naturalists of his time Immanuel Kant articulated an approach to explaining generation. Kant’s account, delineated in his *Kritik der Urteilskraft* (Critique of the power of judgment) (1790), is a combination of Newtonian *non-reductionist mechanism* in explanation, and a concept of natural end comparable to Stahl’s *formal conception of organic bodies.* It consists of two claims: a) in biology only mechanical explanation is explanatory, and b) living entities contain some original organisation, which is mechanically unexplainable. In the nineteenth century this approach influenced naturalists as Müller, Virchow, and Von Baer, in their physiological research. Dissatisfied with a sheer mechanistic or, on the other hand, a sheer teleological approach, they appreciated the Kantian account of mechanical explanation of natural ends. In Germany, in the second halve of the nineteenth century, Ernst Haeckel reopened the debate about abiogenesis, which still continuous.

## Introduction

This paper roots its interest in the question which characteristics are typical for living entities. We approach this issue from a historical perspective. In eighteenth and nineteenth century Europe, both mechanistic^1^ and vitalist approaches to living entities came up. How did some leading scholars in biology deal with the central question of the essential features of life? What historical place had the Kantian account of mechanical explanation of natural ends? What was the influence of this approach among leading naturalists?

In the eighteenth and nineteenth centuries the issue of typical characteristics of living entities was predominantly framed in terms of *biological generation*. With *biology* we refer to all theorizing regarding living entities as living entities (see Zammito, 2018; Gambarotto, 2018 for other definitions). The era discussed roughly covers the period 1790–1876, starting with the publication of Immanuel Kant's *Kritik der Urteilskraft (Critique of the power of judgment)*^2^ (Kant, 1790) and ending with the death of Karl Ernst von Baer. Kant’s *Kritik der Urteilskraft (KdU)* has inspired many naturalists and is still a subject of some discussion among biologists today (e.g. Kauffman, 2019).

The next Sect. (2) delineates the background of a controversy about biological generation. The subsequent Sect. (3) presents the scientific context of the controversy in the eighteenth century. Section 4 describes the emergence of the Kantian view on living entities. Section 5 explicates Kant’s perspective on biology, according to the *KdU*. The subsequent Sects. (6–9) examine the influence of the Kantian account of life on biological research in Europe in the nineteenth century, especially among German scholars. A Sect. (10) with discussion and conclusions completes this paper.

## The rise and impact of the mechanistic world view

In Western philosophical tradition for many centuries, a certain formative force in living entities was held responsible for the development, maintenance, and healing of biological individuals. This living or animating principle that guided living nature had its own existence, independent of and superior to matter (the animistic thesis, cf. Reill, 2005). Since the seventeenth century, however, under the influence of Descartes, a mechanistic world view arose (Dijksterhuis, 1950). In Descartes’s view, all natural phenomena are explainable on the basis of matter and motion alone (cf. Roe, 2010). Descartes himself endorsed a view of gradual formation in embryology, which Sloan (2002) designates *mechanistic epigenesis*. However, in the seventeenth and eighteenth century, mechanistic epigenesis did not convince most embryologists, and—as a result—mechanistic solutions to the problem of generation typically involved some version of preformation of the embryo (cf. Bognon-Küss, 2019; Sloan, 2002). In preformation theories generation is not a gradual formation, but an unfolding (then called ‘evolution’) of parts or germs. According to seventeenth century theories of preformation, generation is merely an unfolding of germs which are pre-existent since creation. In 1670 Claude Perrault propagated a panspermia theory of preformation, according to which numerous pre-existent miniature embryos (germs), containing a complete miniature living entity, float in the air in search of suitable conditions to grow (cf. the *logoi spermatikoi* of the Stoics and the *rationes seminales* of Augustine of Hippo). Subsequently, Malebranche in 1674 formulated another version of preformation theory, called *emboîtement* (nesting). In Malbranche’s version, generation is the unfolding of pre-existent embryo’s (germs), which were enclosed by successive generations (as nested boxes) in the ovum *(ovist emboîtement)* or in the sperm *(animalculist emboîtement).*

In his *Exercitationes de generatione animalium* (1651), the English physician William Harvey had described his precise observations on incubated fertilized chicken eggs. The *Exercitationes* contains the famous dictum (p. 2): “all animals whatsoever are produced from ova”^3^*(omnia omnino animalia (…) ex ovo progigni).* He observed the gradual formation of the chick, part by part. Harvey called it *epigenesis:* “the generation of the chick from the egg is the result of epigenesis, rather than of metamorphosis, and (…) all its parts are not fashioned simultaneously but emerge in their due succession and order” (Harvey & Willis, 1847, p. 336). He assigned the development of the embryo to a *formative faculty*, which proceeds with both consciousness and foresight (p. 399).

In 1684 the German chemist, physician and philosopher Georg Ernst Stahl hypothesized a regulating soul—calling it *Dirigens microcosmicum—*in animated bodies. This soul *(anima)*, in Stahl’s view, is the empirical observable agent, immanent to the organic body, causing its motions. Stahl also distinguished between the material and the formal conception of living bodies (and of their parts, i.e., organs). The material conception of concrete bodies is mechanical and the formal conception of organic bodies is operational or instrumental (Cheung, 2006). In later works, Stahl used the word *organism (organismus)* as a principle of order. He used the word in opposition to *mechanism (mechanismus),* the more or less confused order of aggregates (Cheung, 2006). The concept *organism* betokened complex, rational (although unintentional) self-organization, operating purposefully (Zammito, 2018). Stahl proposed a *“holistic”* concept of the living being (Duchesneau, 1995). Stahl’s *“animism”* influenced European theorizing about living nature. By the mid-eighteenth century *vitalism*, posing vital powers inherent to matter, became the dominant opponent of Cartesian mechanistic natural philosophy (cf. Nicholson, 2010).

It should be noted that the results of research into the properties of organic bodies were limited by the technical possibilities at the time. Due to technical advances of microscopes and better preparation techniques, the cellular structure of all known living entities was discovered and described in the period 1790–1876 (Baker, 1948, 1949, 1953). Developments in chemistry made it possible to conduct more precise analytical research than before into the specific chemical composition of both living and deceased entities (cf. e.g. Berzelius & Wöhler, 1825). New instruments and methods became available to investigate the internal functioning of living entities (Geison, 1969). Thus, research into the phenomena of life increased during that period, but it also became divided over a variety of sub-disciplines.

The rise of a mechanistic world view in seventeenth century Europe provoked a controversy in biology regarding the explanation of generation of living entities in the eighteenth century. Vitalist theories confronted mechanistic theories. Epigenetic theories confronted preformationist theories. There was a lot of confusion about what would count as an adequate explanation; and, subsequently, *what might count as an explanation of generation.*

## Eighteenth century controversy on generation

In this section we will discuss some of the main participants in the controversy on the nature of generation in eighteenth century Europe. The prominent German philosopher Christian Wolff (1679–1754), a rationalist, adopted a conception of the hierarchy of sciences according to which ontology and theology ground natural science. As a consequence, Wolff assigned an explanatory function in natural science to teleology (Van den Berg, 2013). However, to ascribe the purposiveness of living beings to a living God does not provide an explanation, argued Kant (AA 5:395).

Albrecht von Haller (1708–1777), a Swiss physician, is one of the founders of modern physiology. He changed his mind several times regarding the nature of generation. Haller was an empiricist in the Newtonian tradition of *non-reductionist mechanism* which deemed the first cause hidden in an impenetrable obscurity (Zammito, 2018). This mechanism is been distinguished from Cartesian mechanism which explains living entities by matter and initial motion to start things off (Roe, 2010). Haller’s teacher Boerhaave had proclaimed a basic assumption of the Newtonian *experimental philosophy—*to which Haller adhered: “The drive to the ultimate metaphysical and the original physical causes is neither necessary nor useful nor possible for the physician.” (cited in Zammito, 2018, p. 40). Haller initially supported a theory of *animalculist emboîtement*, as had Boerhaave (Detlefsen, 2006). In 1740s, Haller by taking note of experiments with freshwater polyps, became convinced that living entities were engendered from a fluid which thickened and which organized itself little by little, following definite laws (Roe, 1975). In 1752, in the preface to the second part of the German translation of Buffon’s *Histoire naturelle*, Haller described a teleologically directed process capable of generating organic beings by immanent natural causes (Sloan, 2002). In 1758, after experimenting with fertilized eggs, Haller published an *ovist emboîtement* theory of development, stating that all the essential parts of the embryo were in primordial form, but at first transparently, already present in the unfertilized egg (Sloan, 2002). In publications during the 1760s the Swiss scientist Charles Bonnet (1720–1793) elaborated a comparable *ovist emboîtement* view on generation. He regarded generation as the development and growth of pre-existing entities. However, according to Kant, theories that rely on individual preformation (*individuelle Preformation*) do not explain living entities as products of nature (AA 5: 422–425).

In 1749 Georges-Louis Leclerc comte de Buffon (1707–1788) started his impressive *Histoire naturelle* (1749–1767). In it, Buffon challenged some of the basic assumptions of mechanical explanation of life by suggesting additional forces (cf. Roe, 2010). Buffon conceived life as a physical propriety of matter. Organized beings are composed of living particles, *organic molecules*. In organized beings a formative force, an *internal mould,* or *inner form,* is active according to a unique plan generated when the species first appeared (Reill, 2005). A *penetrating force* mediates between the organic molecules and the internal mould. Bognon-Küss (2019) calls it a threefold model in which the penetrating force acts as the efficient cause of organization, the structure (or formal cause) of organization lies in the inner form, while the organic molecules provide the material cause. Buffon articulated a historical species concept in his *Histoire naturelle* (Sloan, 1979). Kant did not agree with the explanatory concept of living matter (AA 5: 392). He considered the concept of living matter (*lebende Materie*) as contradictory and not even conceivable (AA 5: 394). Kant adhered to a historical species concept (AA 5: 422), although a far more restrictive concept than Buffon’s (Sloan, 1979). More important for our subject, Kant—just like Buffon—combined epigenetic and preformationist elements in his concept of generation (AA 5: 423).

Caspar Friedrich Wolff (1733–1794) contested *emboîtement* in 1759 in his thesis *Theoria generationis*. Wolff, one of the founders of embryology, defended an epigenetic theory of development in which he introduced a formative force, which he called *vis essentialis* (cf. Wolff, 1764). It is a nutritive, organizing force (Bognon-Küss, 2019). Wolff sent his dissertation to Haller, and thus began a direct and extended debate which lasted until 1777, the year of Haller’s death (Detlefsen, 2006). The versatile scholar Johann Gottfried Herder (1744–1803) in his *Ideen zur Philosophie der Geschichte der Menschheit (Ideas on the philosophy of human history)* (1785–1786) also distanced himself from mechanism. According to Herder, mechanical laws deal with inanimate matter, vital laws with living matter. He conceived of a new way to perceive the relations between contraries. The history of nature and of humanity demonstrates the interaction between contraries as for example regular development and revolutionary eruption. Contraries are linked by a *mysterious connection*, which he named *harmony* (Reill, 2005). Herder distinguished an *organic force* from a *genetic force*. The genetic force is a general formative principle. It is the cause of epigenetic embryological development, a force that produces organic parts out of the chaos of homogeneous matter (Van den Berg, 2014). Herder’s idea of a plastic force—the genetic force– implies the denial of the epistemological boundary between organized and unorganized beings. This *radical epigeneticism*, which Herder—and even Caspar Wolff—adhered to, involves assuming spontaneous generation (Huneman, 2006; cf. Sloan, 2002; Bognon-Küss, 2019). According to Kant, adherents of a radical epigenetic theory*,* assume absurdly (*ungereimt*) spontaneous generation (*generatio equivoca*) (AA 5: 419). The assumption that unorganized matter can organize itself *(Hylozoism)* does not give a convincingly explanation of generation, according to him (AA 5: 394f). Kant agreed that the embryological observation and description of *epigenesis*, was well attested and studied, among others by C.F. Wolff (cf. Wolff, 1764).

Theoretical biology was a divided field. It contained vitalist and mechanistic theories. Vitalists pursued and expanded Stahl’s ideas. Mechanists like Haller fiercely rebelled against these theories. How to create an evincive unity in this field?

## Kant and Blumenbach: emergence of a new perspective on generation

In 1781 Johann Friedrich Blumenbach (1752–1840), Göttingen's professor of anatomy and comparative zoology, wrote *Über den Bildungstrieb (On the formative drive),* which he thoroughly revised and extended in 1789. Blumenbach (1789) turned against the custom of unrestrained formation of hypotheses concerning reproduction. He combatted various theories of pre-existence, *panspermia* theories and also theories of *emboîtement.* He described experiments and observations, including regeneration in polyps, and concluded that there are no preformed germs. Instead, after the previously raw, unformed generation substance of the organized body has matured and reached its destination, a special lifelong drive *(Trieb)* becomes active. This drive bestows on the germ the power to take on its particular form initially, then preserves it, and, if it is mutilated, to heal when possible (Blumenbach, 1789, p. 24).

Organized bodies *(organisirte Körper)* derive the primary cause of all generation, reproduction, and nutrition from a special inborn formative drive (Blumenbach, 1789, p. 25). Although the formative drive is a life power and as such is inconceivable in inanimate creation, the realm of inanimate nature also has formative powers (*bildende Kräfte*). As an example, Blumenbach mentioned crystallizations, which in their outer form have such a striking resemblance to certain organic bodies, that they give a very appropriate representation, which can somewhat facilitate the imagination of the formation from unformed substances (p. 73).

The phrase *formative drive (Bildungstrieb; nisus formativus),* like words such as attraction *(Attraction)* and weight *(Schwere),* denotes a force whose constant effects have been recognized from experience. Although these are generally recognized forces of nature, their cause remains unknown. The purpose of Blumenbach’s study of these forces is merely to determine their workings more precisely and to trace them back to more general laws (Blumenbach, 1789, p. 25f). These laws appear to exist in perceived regularities such as: the strength of the formative drive is inversely proportionate to the increasing age of the organized bodies (p. 93).Kant also mentioned empirical laws of natural ends in organized beings (AA 5: 382).

Blumenbach distinguished the formative drive from the concept of *vis essentialis* presented by Caspar Wolff (Blumenbach, 1789, pp. 29, 31f). In the second part of his book, Blumenbach determinately challenged Haller's theory of preformation, which he formerly adhered to. Finally, he elaborated his own theory of the formative drive *(Bildungstrieb).* The view Blumenbach articulated is usually called vitalism (cf. Reill, 2005). In distinction from the vitalists figuring in the above section, he asserted to be thoroughly convinced of the great gap that nature has established between living and inanimate creation, between organized and disorganized creatures (p. 71). The formative drive resides only in organized matter. Kant agreed with Blumenbach in rejecting of radical epigenetic theories. Blumenbach influenced Kant, to take more distance to theories of individual preformation (see Sloan, 2002 for an extended historical interpretation). To illustrate the formative drive, Blumenbach described some examples of development and reproduction in plants and cold-blooded animals. He concluded this section with an appeal to the inner feeling of the expert reader that also when considering the reproduction of warm-blooded animals, the belief “of a formative power that shapes the new creature from the unformed reproductive material of the old one,” is much more plausible than the belief “in pre-existing enclosed preformed germs” (Blumenbach, 1789, pp. 82f)*.*

Blumenbach stated that the cause of the formative drive cannot be explained in scientific terms. “Scientific” meaning for Blumenbach Newtonian mechanism. He also stated that the formative drive is an original power. It is a *qualitas occulta*, as Newtonian gravity is. The constant effect of the formative drive must be recognized from observation. In referring to Blumenbach (AA 5: 424), Kant tried to connect with the then current state of affairs in biology.^4^ In this context Kant articulated the view of *the inscrutable principle of an original organization*.

While writing his *Kritik der Urteilskraft*, Kant was in intimate connection with and frequently in strong opposition to leading naturalists and natural philosophers of his time (cf. AA 5: 391ff). The concept of natural purpose with original predispositions, as presented in *KdU,* concerns two capacities of living entities: 1. Preserving the internal purposive organization of the species, 2. modification to external changes. Kant’s approach in *KdU* is an example of combining elements of organism and mechanism. In this way Kant tried to uphold both the peculiarity of life and characteristics of scientific explanation.

## Immanuel Kant on biology

Immanuel Kant (1724–1804) had a restrictive conception of proper science, as a natural consequence of his adherence to a classical ideal of science. Scientific explanations for Kant are, firstly, demonstrations of the proposed facts, giving an account of why something happens (De Jong, 2010; De Jong & Betti, 2010). And secondly, to establish the facts, according to Kant, scientific explanation is properly mechanical explanation (Van den Berg, 2014). Kant adopted Newton’s view that only mathematics based mechanical explanation provides apodictic certainty. But is it possible to explain living entities mechanically? This question is addressed in the second part of Kant’s 3rd Critique (1790), *Kritik der teleologischen Urteilskraft (Critique of the teleological power of judgment)*.

Kant distanced himself from the distinction Descartes makes between humans and animals. Animals, in Kant’s view, are not machines but like humans, they can form representations of their environment. Despite their specific difference, humans belong to the same genus as animals (AA 5: 464). Kant called movement the characteristic of machines. For living entities Kant used the concept of *natural end (Naturzweck).* (AA 5: 369ff). Natural ends have, in addition to a moving power, a self-propagating formative power (AA 5: 374). Natural ends are organized products of nature (AA 5: 376). In a natural end, the parts (as far as their existence and their form are concerned) are possible only through their relation to the whole, and the idea of the whole determines the form and combination of all the parts (AA 5: 373). A natural end is an organized and self-organizing being (AA 5: 374). In natural ends, the connection of efficient causes—i.e., mechanical causality—could at the same time be judged as an effect through final causes (AA 5: 373). By organizing itself, nature is more than merely an analogue of art (AA 5: 374). Kant called the kind of purposiveness, observed in organized beings, internal purposiveness. He defined an organized product of nature as *that in which everything is an end and reciprocally a means*. Nothing in it is in vain, purposeless, or to be ascribed to a blind mechanism of nature (AA 5: 376). The word *“Naturzweck”* has a double meaning in the *KdU*. It indicates a principle of order—even as Stahl used the word *“organismus”*, but it also is a generic name of individuals. Kant did not make use of anything as Stahl’s concept of a regulating soul.

Kant wanted to demarcate biology from metaphysical belief. He discerned among philosophers four attempts to explain natural ends by objective metaphysical principles. First, two systems wherein the purposiveness of nature is conceived as unintentional: the forming of natural ends is either physically explained as an accident *(i.e., System der Kasualität)* or hyperphysically, as a fatality *(i.e., System der Fatalität).* Then, two systems wherein the purposiveness of nature is conceived as intentional: the forming of natural ends is either physically explained as matter acting in accordance with an intention *(i.e., Hylozoism)* (cf. Cudworth, 1743 [1678], Ch. 3), or hyperphysically, as deriving from an original living, intelligent ground of the world-whole *(Urgründe des Weltalls)(i.e., Theism)* (AA 5: 391f). Kant rejected them all for not being explanatory. He concluded that the generation of natural ends can only be interpreted as being established through a supreme understanding *(einem obersten Verstande)* as the cause of the world. But this conclusion is merely a heuristic teleological principle. It does not explain anything (AA 5: 395).

In analogy with causality according to ends—as we perceive in art, Kant conceived of nature as technical through its own capacity, ascribing agency *(Wirkungsart)* to it (AA 5: 360). By regarding living entities as *organized* Kant rejected reductionist mechanistic theories as e.g. Cartesian mechanism (cf. Roe, 2010).

Kant has considered the hypothesis of the generation of organized beings (*organisierte Wesen)* out of the *maternal womb (Mutterschoss)* of the earth and from each other in an ascending degree of complexity. He wrote in a note that a hypothesis of this sort can be called “a daring adventure of reason.” Moreover, according to Kant, whoever proposes the production of organized beings by the earth, must attribute to this universal mother an organization purposively aimed at all these creatures, for otherwise the possibility of the purposive forma of the products of the animal and vegetable kingdoms cannot be conceived at all (AA 5: 419).

According to Kant, natural ends are not formed as repeated instantaneous miracles (occasionalism), for then the concept of the natural is entirely lost (AA 5: 422). Natural ends are also not formed as educts,^5^ by individual preformation and wrapped in an original ancestor. They are formed by the system of generatings as product, called the system of *epigenesis*, or the system of *generic preformation* (AA 5: 422f). In the concept *product,* Kant proposed an alternative model to growth by aggregation. All generation that we know is *generatio homonyma—*in its organization itself the product is homogeneous with that which generated it (AA 5: 419f). The forming of entities by a *formative power*, i.e., epigenetic formation, means the generation of offspring by a progenitor in accordance with its internally purposive predispositions *(inneren zweckmäßigen Anlagen)* (AA 5: 423).

Kant posed that his teleological concept of *natural end* has no metaphysical implications (AA 5: 392, 395). It is merely a regulative principle (AA 5: 405)—that is, an heuristic principle. Treating it as constitutive would be affirming the reality of causation by intelligence in nature (AA 5: 383). Science should limit itself to mechanical explanations and not make statements about metaphysical questions. The question of the origin of the inner purposiveness of living entities, is such a metaphysical question (AA 5: 383). In answering the question of the relationship between mechanical causality and ideal causality, Kant refers to an unknowable supersensible ground of nature (AA 5: 422) and to the inscrutable principle of an original organization (AA 5: 424). Kant’s teleological concept is not meant as an explanation, it only has a heuristic value to identify the object of biological research (AA 5: 411). To be in accordance with his *Critical philosophy,* Kant could in the *KdU* not use dogmatic assertions, i.e., objective metaphysical principles (AA 5: 395), so his concept of natural end must be a principle *as if.* We should treat natural ends *as if* they were the product of an idea, a plan that governed their operations, a plan generated by an *Intellectus archetypus,* a divine understanding (AA 5: 408). According to Kant, this is a helpful heuristic that enables us to push mechanical explanation as far as we can. By regarding the concept of a purposiveness of nature as a merely *regulative principle* (AA 5: 197) Kant excluded rationalist *(theistic)* theories.

Generation and also the functioning of living entities, must be explained mechanically, according to Kant. In the natural sciences, teleological concepts do not belong to the *explanans* but to the *explanandum*, they have no explanatory value (AA 5: 360). Teleology only has a place in a description of nature, explanations always have to be mechanical (AA 5: 417). In this statement Kant differs clearly from Stahl. In explaining living nature, the investigator has to pursue mechanical explanation as far as possible. But there will never arise a Newton who could make comprehensible even the generation of a blade of grass according to natural laws that no intention has ordered (AA 5: 400).

*We conclude: 1. The account Kant articulated in KdU for explaining generation combined (a.) the concept of natural end which is—as being a principle of order—comparable to Stahl’s formal conception of living bodies (see Sect. 2), and (b.) the view that scientific explanation is to be perceived as Newtonian non-reductionist mechanism. 2. Kant’s approach can be properly summarized as “mechanical explanation of natural ends.”* His approach consists of two claims: a) in biology only mechanical explanation is explanatory, and b) living entities contain some original organisation, which is mechanically unexplainable. *We hypothesize that this approach was used by some leading researchers in physiology—physiology taken in a wide sense—during the nineteenth century.*

Though more explicit in its articulation, Kant’s approach has closely related precursors—cf. Wolfe and Terada (2008), and Wolfe (2014). Noting that Kant influenced scientists of his time and thereafter does not necessarily imply that Kant’s *KdU* has been the main influence for them using this approach. An important example of Kant’s influence on outstanding researchers is Johannes Müller, to whom we now turn.

## Development of physiology

Johannes Müller (1801–1858) was one of the prominent physiologists of his time. His famous *Handbuch der Physiologie des Menschen (Handbook of human physiology)* (2 volumes) was printed in the 30 s and 40 s in many editions and was also translated into English: *Elements of physiology* (Müller & Baly, ). After a London edition, in 1843, an American abbreviated edition, also titled *Elements of Physiology* (Müller et al., 1843), was published.^6^ Müller started the *Prolegomena* of his *Handbuch* in this way (Müller, 1835, p. 1):

Physiology is the science which treats of the properties of organic bodies, animal and vegetable, of the phenomena they present, and of the laws which govern their actions. Inorganic substances are the objects of other sciences, - physics and chemistry. In entering upon the study of physiology, the first point to be ascertained regards the distinctions between these two great classes of bodies – the organic and the inorganic, - and the following questions suggest themselves for discussion. Do organic and inorganic substances differ in their material composition? and since the phenomena presented by these two classes are obviously so different, are the forces or principles on which they depend, also different; or are the forces which give rise to the phenomena of the organic kingdom merely modifications of those which produce physical and chemical actions?^7^

Trivializing the in vitro production of urea from inorganic ingredients—realized in 1828 by Friedrich Wöhler (Müller et al., 1843, p. 15), Müller stated that “organic matter is never produced spontaneously” (p. 26). The basic force for organic phenomena is the *vital force* (Müller, 1835, p. 18):

In the production of Infusoria [term used in the nineteenth century to indicate an order of unicellular animals, WB & HJ] there is no new formation of organic matter; the previous existence of organic beings is presupposed. Organic matter is never produced spontaneously. Plants alone seem to have the power of generating ternary or organic compounds from binary or inorganic compounds; while animals are nourished only by organic matter, which they cannot generate from binary compounds, and consequently their existence presupposes that of the vegetable kingdom. How organic beings were originally produced, and how organic matter became endowed with a force which is absolutely necessary to its formation and preservation, but which is manifested only in it, are questions beyond the compass of our experience and knowledge to determine. The difficulty is not removed by saying that the organic force has resided in the organic matter from eternity, as if organic force and organic matter were only different ways of regarding the same object: for, in fact, the organic or vital phenomena are presented only by a certain combination of the elements; and even organic matter, itself susceptible of life, is reduced to inorganic compounds as soon as the cause of the vital phenomena, namely, the vital force, ceases to exist in it. This problem, however, is not a subject of experimental physiology, but of philosophy. Conviction in philosophy and in natural science have entirely different bases; the first suggestion here, therefore, is, not to be led away from the field of rational experiment. We must be content to know that the forces which give life to organic bodies are peculiar, and then examine more closely their properties.^8^

The vital force is an organizing force, acting in accordance with rational laws *(vernünftigen Gesetzen),* the final cause *(Endursache)* of organic bodies, argued Müller. It is a creative force which changes matter in a purposeful way. An organic being is the factual unit of organic creative force and organic matter (Müller, 1835, p. 25). Therefore, an organism *(Organismus)*^9^ forms a whole (p. 19):

The manner in which their elements are combined, is not the only difference between organic and inorganic bodies; there is in living organic matter a principle constantly in action, the operations of which are in accordance with a rational plan, so that the individual parts which it creates in the body, are adapted to the design of the whole; and this it is which distinguishes organism. Kant says: “the cause of the particular mode of existence of each part of a living body resides in the whole, while in dead masses each part contains this cause within itself.”^10^

Did Müller, in contrast to Kant, consider the teleological principle as explanatory? He conceptualized a *vital force* which is an organizing force expressing itself by a rational law. In formulating this concept, he is indebted to Stahl’s concept of *soul.* Müller interpreted the soul *(anima),* spoken of by Stahl (see Sect. 2), as the organising power or principle which manifests itself in conformity with a rational law, and he endorsed this concept (Müller, 1835, p. 24; cf. Müller et al., (1843), p. 32). It did not hinder him—unlike Stahl—to seek to explain organisms mechanically. For the breakthrough of the mechanical approach *(der mechanischen Richtung)* in physiology, Müller has done more than any other physiologist before him (Haeckel, 1866^a^, p. 94). How could this happen? Müller’s “animism” means he did not regard living entities to be merely an aggregate of mechanisms (cf. Müller, 1835, p. 23, 30). Yet, reading Kant (cf. Müller, 1835, p. 19) gives this *“vitalist”—*as Haeckel called him—a theoretical basis for mechanical explanation in physiology. His explanations got a prominent place in nineteenth century physiology.

## Cell theory

In the nineteenth century, research in the internal organization of organisms made progress (cf. Mendelsohn, 1965). In 1838 Matthias Schleiden published his *Beiträge zur Phytogenesis.*^11^ In it he focused attention on the nucleus and the cell wall as characteristic components of the cells of spermatophytic plants. Shortly afterwards Theodor Schwann (1810–1882), his friend, an assistant of Müller, published his *Mikroskopische Untersuchungen (Microscopical researches)* (Schwann, 1839). In its first part, he generalised Schleiden’s observations on the basis of his own observations and stated that one common principle of development exists for all organic parts of both plants and animals, namely the cell. He suggested the term *cell theory (Zellentheorie)* for this theory (Schwann, 1839, iv and 197).

In the last part of his treatise (from page 220), Schwann speculated about the consequences of his *cell theory*, giving this latter part the name *theory of the cells (Theorie der Zelle).* Schwann himself did not regard it a part of his *cell theory*.^12^ According to his *theory of the cells*, the fundamental organic processes take place at the level of the cell and not at the level of the entire organism. Every elementary part (every cell) has an autonomous life (p. 228). The question of the fundamental force of organized bodies is reduced to the question of the fundamental force of individual cells (p. 229). Schwann further stated (p. 222):

The adaptation to a purpose which is characteristic of organized bodies differs only in degree from what is apparent also in the inorganic part of nature; and the explanation that organized bodies are developed, like all the phenomena of inorganic nature, by the operation of blind laws framed with the matter, cannot be rejected as impossible. Reason certainly requires some ground for such adaptation, but for her it is sufficient to assume that matter with the powers inherent in it owes its existence to a rational Being.^13^

Organized bodies (organisms) are formed by merely physicochemical regularity (p. 226). Schwann summarized (p. 257):

The view then that organisms are nothing but the form under which substances capable of imbibition crystallize, appears to be compatible with the most important phenomena of organic life, and may be so far admitted, that it is a possible hypothesis, or attempt towards an explanation of these phenomena. It involves very much that is uncertain and paradoxical, but I have developed it in detail, because it may serve as a guide for new investigations.^14^

Müller in his *Handbuch* commented on a consequence Schwann inferred in the treatise. Does every cell contain the power to generate a whole organism? Müller agreed with him as far as the *lower* biological beings are concerned but saw major problems regarding the *higher* animals. The *theory of the cells* places too much emphasis on the potentiality and equality of the body cells, ignoring the influences of the organism as a whole on individual cells and differences between cells, according to Müller (Müller, 1840, p. 614ff).

Around the middle of the nineteenth century a profound change occurred in the beliefs of biologists about the way in which cells multiply (Baker, 1953). In the 1850s, Robert Remak (1815–1865) and Rudolf Ludwig Carl Virchow (1821–1902), students of Müller, both examined the cell theory of Schwann. In 1859 – after previous related papers by Remak and by Virchow, Virchow stated the cellular origin of every single cell, and supplemented the cell theory of Schwann accordingly. The initial cell theory was renamed by Virchow *theory of free cell formation (Theorie der freien Zellenbildung)* (Virchow, 1859, p. 9) to discriminate it from his own supplementary cell theory. He concluded in his *lectures* on *cellular pathology* (Virchow, 18591859, p. 25): “Where a cell arises, there a cell must have previously existed *(Omnis cellula e cellula).*”

In an 1858 essay, translated as *On the mechanistic interpretation of life,* Virchow discriminated between his “mechanistic interpretation of life” and *materialism* (Virchow & Rather, 1958, p. 108). He also distinguished his view from *spiritualism* (p. 115). Virchow tried to avoid transcendent questions. In his opinion th*e “*biologist first seeks for the plan, or, as we may otherwise say, for the law. The next question, then, after the law has been found, is how the law or plan has been carried out, not who made it*”* (p. 114). Considering organic and inorganic creation both, Virchow stated: “Everywhere there is mechanistic process only, with the unbreakable necessity of cause and effect. The plan is in the body, the ideal in the real, the power in the material.” (p. 115).

Cell theory signified an important step for biological theorizing. The intracellular organization, especially the role of the nucleus, gradually obtained more clarification. Virchow discussed biological regularity: “Life is cell activity; its uniqueness is the uniqueness of the cell. The cell is a concrete structure composed of definite chemical substances and built up according to a definite law” (p. 106). Mechanical explanation is required: “As particular, as peculiar, and as much interiorized as life is, (…), so little is it withdrawn from the rule of chemical and physical law” (p. 107). When, regarding our comprehension of *unusual* phenomena, Virchow remarked: “we comprehend it only in a mechanistic sequence of cause and effect. For the human spirit is incapable of any other kind of comprehension” (p. 111f). Here we note the influence of Kant. It was Virchow’s approach of biological research as aiming at mechanical explanation of purposive entities that brought cell theory to its prime. The cell plasm became another important topic in nineteenth century discussion on generation. It provoked new theorizing about the origin of living organisms.

## Protoplasm theory

The phrase *spontaneous generation* can refer to both *heterogenesis* and *abiogenesis*. *Heterogenesis* can mean that micro-organisms originate from dead organic material or that parasitic worms arise from other living organisms (Farley, 1972^b^). *Abiogenesis,* also called *generatio aequivoca*, refers to the generation of living entities from inanimate (“raw”) matter. Books like *Cellularpathologie* (Virchow, 1859) show that the discussion about *heterogenesis* came to an end in the nineteenth century. *Heterogenesis* was no longer believed to occur. Belief in the possibility of *abiogenesis* had almost disappeared in the second half of the eighteenth century. The *Encyclopaedia Brittanica* (1771) noted under the lemma *equivocal generation*: “this kind of generation is now quite exploded by the learned” (quoted in Farley, 1972^a^) and Blumenbach wrote in his *Handbuch der Naturgeschichte (Handbook of natural history)* about *generatio aequivoca* that he deemed it to be thoroughly refuted *(gründlich widerlegt zu seyn)* (Blumenbach, 1779, p. 21).

The discussion of *abiogenesis* was given a new impetus by the appearance of Darwin's *Origin of Species* (Darwin, 1859; see Farley, 1972^a^). Darwin explained the diversity perceived in living nature through natural selection of heritable variation in *striving organic beings*. This raised the question whether a natural explanation is possible for *the origin* of living entities. Ernst Haeckel picked up this gauntlet, developing an idea from Schwann’s theory of the cells. In England and Germany in the 1860s and 1870s a heated discussion took place about protoplasm (cf. Geison, 1969). We will focus mainly on some developments in scientific discourse on protoplasm in the German states*.*

In the 1840s and 1850s, a lot of research took place on cell plasm (cf. Baker, 1949; Geison, 1969). The German botanist Hugo von Mohl criticized the degree to which Schleiden and Schwann exalted the cell wall. In an 1846 article he defined the word *protoplasm* clearly and emphasized its relation to the nucleus (Mohl, 1846). In 1850 Ferdinand Cohn argued that “the *protoplasm* of Botanists, and the contractile substance and *sarcode* of Zoologists, if not identical, are at all events in the highest degree analogous formations,” and that this substance “must be regarded as the prime seat of almost all vital activity” (Cohn, 1853 [1850], p. 534f). The Austrian botanist and physiologist Franz Unger emphasized the conformity in form, composition and action *(Form, Beschaffenheit und Wirksamkei*t*)* of protoplasm between lower animals and plants (Unger, 1855). *“*A cell is a little blob of protoplasm, containing a nucleus,” concludes the German microscopic anatomist Max Schultze (1861, p. 11). Cell theory and protoplasm theory both, and particularly together, suggest the importance of the material composition of cells.

The gifted versatile scientist Ernst Haeckel (1834–1919), student of Müller and for some time assistant to Virchow (Haeckel, 1866^a^, p. xvii), made, among other things, a careful study of the protozoa phylum *Radiolaria* (Haeckel, 1862). He became convinced that mechanical reasoning suffices for explaining life. In the first part of his *Generelle Morphologie* (1866a), Ernst Haeckel dedicated a section to “*Teleology and Causality (Vitalism and Mechanism)”*. In his introduction of this subject, he combined two quotes, from Müllers’s *Handbuch* (Müller, 1840, p. 505 and Müller, 1844, p. 23).

Haeckel (1866^a^, p. 94) wrote:

A piece of mechanism is formed in accordance with an idea held in view by the artificer, this idea being the purpose for which it is intended. An “idea” also regulates the structure of every organism, and of each of its component organs. In the former case, however, the ruling idea exists external to the artificial mechanism, namely, in the mind of the artificer; while the idea, which is the cause of the harmony of organic bodies, is in action in the organism itself, exerting in it a formative power **unconsciously, and in obedience to determinate laws.** For the purposefully producing cause of the organic body has no choice, and the realization of a single plan is its **necessity; rather, for this producing cause applies: producing for adaptation and producing in accordance to determinate law are one and the same thing.** Therefore, the organising principle cannot be compared with mental consciousness; one should not compare its blind necessary activity with the forming of a concept. Organism is the result of the union of the organic creative power and organic matter.^15^

The first quote (“A piece of mechanism…” until “…one and the same thing”) focuses our attention on the formative power, shaping by necessity. The second quote, the remaining lines—added with the conjunction “therefore,” focuses our attention on the difference between this organizing principle and mental consciousness (or mind). The blindness of the formative power, its activity according to determinate laws, receives emphasis. This power is *less* than mental consciousness. But did Haeckel do justice to the view of Müller, who had died in 1858, by presenting Müller’s words in this way? In the second quotation, Haeckel omits some lines and words present in Müller’s *Handbuch*, shown below in italics*.* Müller (1844, p. 23) had written:

The organizing principle, cannot be compared with mental consciousness; one should not compare its blind necessary activity with the forming of a concept. *Mind can generate no organic products, it can merely form conceptions; our ideas of the organized being are mere conscious conceptions of the mind. The formative or organizing principle, on the contrary, is a creative power modifying matter, blindly and unconsciously, according to the laws of adaptation.* Organism*, or the organized state,* is the result of the union of the organic creative power and organic matter.^16^

In Müller’s rendering, not only the blindness of the formative power gets attention, but especially its effectiveness. This power, in generating organised beings, is *more* than our ideas of the organised being, which are *mere* conscious conceptions of the mind. The formative power, however, modifies matter purposefully into an organic product (a full-grown organism).

Haeckel praised Müller as a researcher who had done a great deal for the breakthrough of the mechanical approach in physiology (cf. Section 6), but regretted his supposed *vitalism*. While opposing vitalism, Haeckel simultaneously rejected a *teleological* approach. According to Haeckel, Müller and many other researchers of nature were entangled in a dualistic conflict. Haeckel pointed a way out (Haeckel, 1866^a^, p. 95):

If, as Müller says, goal-oriented causation and necessary causation in the active cause in the organism is one and the same, then the goal-oriented *causa finalis* coincides with the mechanical *causa efficiens,* then the former abolishes itself to submit to the latter, then it is acknowledged that the mechanical concept of the organisms is the only correct one.

Haeckel opposed dualism with monism: the knowable world forms a unity (a monon), subject to one and the same mechanical law. Living bodies have ever been generated by *Urzeugung,* i.e. (Haeckel, 1866^a^, p. 170):

…a first formation of living bodies through the primal, inherent forces of matter, which act with absolute necessity according to determinate law.

In volume two of his *Generelle Morphologie* Haeckel wrote under the heading “*Different ways of Generation”* (Haeckel, 1866^b^, p. 33):

All generation, i.e., all origination of organic individuals, is either spontaneous generation *(generatio spontanea)* or parental generation *(generatio parentalis).*

Within *generatio spontanea*, Haeckel distinguished between autogeny *(Autogonie)* and plasmgeny *(Plasmogonie).* Monera *(Moneres)* (from Greek; “simple ones”), i.e., prokaryotes, could arise from a formation fluid (*Bildungsflüssigkeit*) with only inorganic compounds *(Autogonie).* They can also arise from a formation fluid with organic compounds *(Plasmogonie).* Haeckel wrote (1866b, p. 33):

The parentless generation in an “inorganic” formation fluid, the **autogeny or self-generation**, is the mode of *generatio spontanea* with which the organic life on the previously inanimate earth crust must have started at any time. As we do not know it from observation, we do not know whether it currently persists. But necessary is the important deductive conclusion that once organic individuals of the simplest kind (Monera) must have originated directly through the coming together of simple (inorganic) compounds to form complexes (protein-like carbon compounds).^17^

Haeckel characterized Monera (Haeckel, 1866^b^, p. xxii):

We name Monera all **completely structureless and homogeneous organisms**, which consist only of a piece of plasma (a mucilaginous protein compound) that simply feeds through endosmosis and propagates through schizogony or sporogony.^18^

Haeckel (1866^a^, p. 154) discerned in any natural body *(Naturkörper),* i.e., *an organic or inorganic individual* (p. 149), two forming forces *(formbildende Kräfte)*: the *internal formative drive (inner Bildungstrieb),* i.e., the material composition, and the *external formative drive (äussere Bildungstrieb),* i.e., the sum of the forces which affect the natural body from the environment, resulting in adaptation. The internal formative drive is the sum of the forces in the atomic composition of a natural body. The external formative drive induces modification of the internal material composition (Haeckel, 1866^a^, p. 154ff; cf. Darwin, 1859; see also Russell, 1916). On the origin of the atomic composition of organic natural bodies, Haeckel articulated the view that the forces of matter, i.e., mechanical forces, suffice to explain the original composition of some first ancestors. Haeckel’s assumption set the stage for origin of life inquiry after him.

## New embryological insights

In the nineteenth century, the Baltic German Karl Ernst von Baer (1792–1876), professor at Königsberg and later at St Petersburg, is accredited with achieving important advances in the field of embryology. Von Baer was a scientist active in scientific disciplines like zoology and anatomy, and also in anthropology and geography (See e.g. Stieda, 1878; Tammiksaar & Kaavere, 1999). Oppenheimer (1990) writes of him: “He performed the most important work in embryology that has ever been done; he started embryology off in the direction which it still follows.” In the 1820s, Von Baer discovered the mammalian egg. In 1827, after anatomical observation in dogs, he wrote the treatise *De ovi mammalium et hominis genesi (On the genesis of the ovum of mammals and of man)* (Von Baer, 1827)*.*^19^ In it, he answered one of the most challenging questions in mammalian biology, by describing how he discovered the mammalian egg (Cohen in Von Baer & O’Malley, 1956).

Although in his 1827 treatise *On the genesis of the ovum of mammals and of man,* Von Baer once mentioned a *formative force (vis formativa),* he did not use concepts like *formative force (Gestaltungskraft)* or *vital force (Lebenskraft)* in his *Entwicklungsgeschichte* (Von Baer, 1828, 1837). In 1876 Von Baer devoted a critical discussion to the concept *vital force.* He wrote that *vital force* is “an attempted concealment of the task that we want to resolve” (Von Baer (1886^2^, p. 187). A clearly Kantian approach of explanation.

Lenoir has made plausible that Von Baer was convinced that the biologist should employ a mechanistic framework of explanation wherever it is relevant. In embryology, Von Baer directed his attention toward identifying the irreducible organizational forms postulated by Kant and to identify concretely the associated *predispositions (Anlagen)* (cf. Lenoir, 1988). The mammalian egg reveals correspondences in the formation process among the different classes of animals (Vienne, 2015). Von Baer concluded: “Every animal which springs from the coition of male and female is developed from an ovum, and none from a simple, formative liquid” (Von Baer, 1827).^20^ He broke with the prevailing view that in mammals the embryo was formed from a female generative substance (Vienne, 2015).

In 1828 the first volume of his *Über Entwicklungsgeschichte der Thiere (On the developmental history of animals)* was published. In an introductory word, in which he also refers to the pioneering work of Caspar Friedrich Wolff*,* he wrote (Von Baer, 1828, p. xxii):

I would be content, if it would be considered my contribution, to have proved, that the mode of development will be determined by the type of organisation.

Von Baer agreed with Georges Cuvier that animals may be classified into four basic types: the Vertebrata, the Articulata, the Mollusca, and the Radiata (Von Baer, 1828, pp. vii, 225, 259). Von Baer in his *Entwicklungsgeschichte,* rejected recapitulation theories^21^ and preformationist theories of development. In it he formulated, among other things, his famous four laws of embryology. He stated (Von Baer, 1828, p. 225) that the individual development is a progress from a more general form to a more special form, whereas recapitulation theories claim in embryo development the transition from one specific form to another.

Von Baer supposed an inner connection between all natural phenomena. His entomological investigations taught him that the perception of distance and time will vary greatly between organisms that differ vastly in size and lifespan (cf. Von Baer, 1864, pp. 237–284, in particular p. 283). He stated that the inner life of an animal can run faster or slower in a same period of outer time, and that this inner life is the basic measure with which we measure time when observing nature (Von Baer, 1864, pp. 273f). Von Baer called an organism a machine, a mechanical device (Von Baer, 1886^2^, p. 188). Natural laws are the constant factor (Von Baer, 1864, pp. 268f). In some sense one is allowed to compare an organism with a clockwork. The organism, however, forms itself according to an internal rhythm (Von Baer, 1886^2^, pp. 179f). The individual parts of organisms are built according to the type and rhythm of the corresponding life process and by its effectiveness, so that they cannot serve another life process (Von Baer, 1886^2^, pp. 180f).

Von Baer saw in nature a harmonious artwork *(harmonisches Werken)* (Von Baer, 1886^2^, p. 181). By *harmonious* Von Baer meaned a regulated reciprocal relationship between organisms. (Von Baer, 1886^2^, p. 228). The organization in time matters. Von Baer tried to clarify that mechanical law alone does not explain nature. The individual tones don’t form the melody, he stated, rather the individual tones are being produced in the sequence necessary to make the melody perceptible. A melody, in a sense, consists of a sequence of tones, i.e., vibrations of the air, but not merely of a sequence of tones. The melody does not just arise from these individual physical events, for another constellation of these tones would not result in this music, but only in a jumble of sounds (Von Baer, 1864, p. 280). Taking aim particular at Darwin, Von Baer held that chance cannot produce anything permanent, only disturb (Von Baer, 1886^2^, p. 229).

Von Baer regarded nature as an eternal becoming (*ein ewiges Werden*) (Von Baer, 1886^2^, p. 231). Every organism demonstrates goal-directedness *(Zielstrebigkeit).* For nature as a whole, Von Baer sometimes used the term purposefulness *(Zweckmässigkeit)* (p. 224). Von Baer left the choice to the reader, and formulated: “the whole of nature operates rationally” or “the whole of nature is rational” (p. 229). Von Baer viewed nature as directed toward an end. This concerns the whole of nature, animate and inanimate, and also individual living entities. He concluded that all organic development is thoroughly purposive, because the descendants should reach the organization of the producing entities. The result of the development is, therefore, determined beforehand (p. 234). Von Baer viewed in nature a unity which is impenetrable (p. 181). Earlier he had stated “that life (…) does not have to be explained from something else, but has to be understood and comprehended by itself” (Von Baer, 1837, p. 3). Von Baer pursued mechanical explanation, but at the same time envisioned nature as goal-directed, which he considered as mechanical inexplicable.

## Discussion and conclusions

Kant ingeniously combined organism and mechanism in his philosophy of living nature. His approach can be characterized as combining Newtonian *non-reductionist mechanism* in explanation with a concept of natural end which is—as being a principle of order—comparable to Stahl’s *formal conception of organic bodies*. Kant’s approach contains elements of both epigenetic and preformationist theories by equating *epigenesis* with *generic preformation*. His account of biology can be properly summarized as *mechanical explanation of natural ends*.

It turns out that the Kantian account of life, as articulated in the *KdU*, influenced scholars. It helped scholars to describe biological phenomena, and to explain them scientifically. It is not easy to reconstruct eighteenth and nineteenth century considerations. Zammito’s (2018) recent monography delivers a competent and fascinating, but—with regard to “several of the undisputed titans of German life science of the early nineteenth century” (p. 340)—incomplete picture. Our study demonstrates that the approach to generation articulated in Kant’s *KdU* had an impact on biologists’ reflection on development in nineteenth century. Some very productive scholars in physiology—and related disciplines—in the nineteenth century, Müller, Virchow, and Von Baer, used an approach in explaining generation comparable to the approach in the *KdU*. With this conclusion we side with Lenoir’s (1989 [1982]) claim concerning the existence of a “teleomechanist program” in nineteenth century Germany, associated with Kant’s *KdU*. Our research has demonstrated that some important discoveries are due to this approach.

Using the same explanatory approach does not necessarily imply to have an identical view on the question ‘What is life?’. In distinction to Kant in the *KdU*, Müller conceptualized in his *Handbuch* a *vital force* resembling the *anima* hypothesized by Stahl (see Sect. 6). He regarded questions regarding the origin of this force “not a subject of experimental physiology, but of philosophy,” and concluded: “We must be content to know that the forces which give life to organic bodies are peculiar, and then examine more closely their properties.” In this way he could make use of Kant’s approach in the *KdU*. We furthermore contend that Müller was not a vitalist, for he did not conceive of life as a property of matter, but differentiated between *organic creative power* and *organic matter* (see Sect. 8).

How mechanical phenomena can result in biological phenomena, is a fundamental question in origin of life research. Haeckel took an essential step. He elaborated the above-mentioned statement from Schwann’s *theory of the cells:* “The adaptation to a purpose which is characteristic of organized bodies differs only in degree from what is apparent also in the inorganic part of nature; and the explanation that organized bodies are developed, like all the phenomena of inorganic nature, by the operation of blind laws framed with the matter, cannot be rejected as impossible” (Schwann, 1839, p. 222). For Müller, teleological descriptions and mechanical descriptions of life phenomena are complementary—as it was for Kant. *Natural ends* display—besides mechanical regularity—biological regularity. Ginsborg (2001; cf. 2014) makes plausible that the way Kant regarded biological phenomena as lawlike, given their contingency with respect to the laws of matter, was to regard them as *governed by normative rules*. Mechanical causality explains life phenomena, but it does this to a certain extent—not exhaustively. Virchow deduced all explanation of life phenomena to mechanical explanation of functioning cells. Virchow and Müller both regarded mechanical explanation to be the explanation of the functioning of an organised entity. Von Baer sided with them. For Haeckel mechanical explanation *included* the emergence of the original, adapted organisation. This view poses a challenge for origin of life research.

## Footnotes

1 In this paper the terms ‘mechanistic’ and ‘mechanical’ cannot be sharply distinguished from each other.

2 References to Kant’s writing are to the Akademie Ausgabe (AA). *KdU* is in volume 5. English quotations are from Kant et al. (2000).

3 English translation by Willis.

4 Debate on the relationship between Kant and Blumenbach can be found in e.g. Larson (1979), Lenoir (1980), Richards (2000), Zammito (2003) and (2012), Huneman (2006), Van den Berg (2014), and Gambarotto (2018). In our view, to safeguard the non-explanatory character of formative drive (and other vital forces)—mentioned by Blumenbach in living entities—was the issue at stake for Kant.

5 Educt is a chemical concept, signifying**:** a substance separated from material in which it already existed—distinguished from product*.* https://www.merriam-webster.com/dictionary/educt (Accessed 23 September 2021).

6 For translating parts of the *Handbuch* into English we have made use of the work done by Baly.

7 English translation by Baly. See Müller and Baly (1838), p. 1.

8 English translation by Baly. See Müller et al. (1843), pp. 26f.

9 Since around 1800 the double occurrence of the word *“organism” (Organismus; pl. Organismen)* both as a principle of order and as a generic name of individuals characterizes German biology (cf. Cheung, 2006).

10 English translation by Baly. See Müller et al. (1843), p. 27.

11 …in the second issue of t*he Archiv für Anatomie**, **Physiologie und Wissenschaftliche Medicin*, then commonly referred to as *Müller’s Archiv*.

12 So also Baker (1948).

13 English translation by Smith. See Schwann and Smith (1847).

14 Idem.

15 *“Ein mechanisches Kunstwerk ist hervorgebracht nach einer dem Künstler vorschwebenden Idee, dem Zwecke seiner Wirkung. Eine Idee liegt auch jedem Organismus zu Grunde, und nach dieser Idee werden alle Organe zweckmässig organisirt; aber diese Idee ist ausser der Maschine, dagegen in dem Organismus, und hier schafft sie m i t  N o t w e n d i g k e i t  u n d  o h n e  A b s i c h t. Denn die zweckmässig (wirkende) wirksame Ursache der organischen Körper hat keinerlei Wahl, und die Verwirklichung eines einzigen Plans ist ihre  N o t w e n d i g k e i t;  v i e l m e h r  i s t  z w e c k m ä s s i g  w i r k e n  u n d  n o t h w e n d i g  w i r k e n  i n  d i e s e r  w i r k s a m e n  U r s a c h e  e i n  u n d  d a s s e l b e.  Man darf daher die organisirende Kraft nicht mit etwas dem Geistesbewusstsein Analogen, man darf ihre blinde nothwendige Thätigkeit mit keinem Begriffbilden vergleichen. Organismus ist die factische Einheit von organischer Schöpfungskraft und organischer Materie.”* Accentuation by Haeckel. Müller omits the word placed in brackets. For translating into English we have made use of the work done by Baly, which we have supplemented. See Müller and Baly (1842), p. 1333 and (1838), p. 25.

16 *Man darf daher die organisirende Kraft nicht mit etwas dem Geistesbewusstseyn Analogen, man darf ihre blinde nothwendige Thätigkeit mit keinem Begriffbilden vergleichen. Unsere Begriffe vom organischen Ganzen sind blosse bewusste Vorstellungen. Die organische Kraft dagegen, die Endursache des organischen Wesens, ist eine die Materie zweckmässig verändernde Schöpfungskraft. Organisches Wesen, Organismus ist die factische Einheit von organischer Schöpfungskraft und organischer Materie.* Idem in Müller (1835), p. 25. The English translation of the intended lines and words is by Baly. See Müller and Baly (1838), p. 25.

17 Accentuation by Haeckel.

18 Idem.

19 For a translation into English: see Von Baer and O'Malley (1956).

20 English translation by O’Malley.

21 In 1833/1834 Joh. Müller endorsed such a theory, but in 1835 no longer. See Von Baer (1886^2^), p. 243.
